# Editorial: Autism: the movement (sensing) perspective a decade later

**DOI:** 10.3389/fnint.2025.1634265

**Published:** 2025-06-19

**Authors:** Elizabeth B. Torres, Brittany G. Travers, Jonathan T. Delafield-Butt, Ashok Srinivasan

**Affiliations:** ^1^Psychology, Computer Science, Cognitive Science, Rutgers, The State University of New Jersey, New Brunswick, NJ, United States; ^2^Occupational Therapy Program in the Department of Kinesiology, University of Wisconsin-Madison, Madison, WI, United States; ^3^Waisman Center, University of Wisconsin-Madison, Madison, WI, United States; ^4^Laboratory for Innovation in Autism, University of Strathclyde, Glasgow, United Kingdom; ^5^Strathclyde Institute of Education, University of Strathclyde, Glasgow, United Kingdom; ^6^The Autism Impact Fund, San Francisco, CA, United States

**Keywords:** autism, motor, kinesthetic reafference, digital biomarkers, mobile technologies and applications, physical exercise, neurological music therapy (NMT), Feldenkrais Method^®^

## Introduction

In 2013, I had the honor to co-Edit with the late Anne Donnellan, the Research Topic (RT) Autism: the Movement Perspective. Anne would be proud to see the high impact of our former RT and the fast-growing impact of its renovated current version, a little over a decade later. We thank you, Professor, Mentor, Trailblazer, and Mother of our growing field, Anne Marie Donnellan, by opening our RT with self-advocate Dr. Kapp's (a) opinion piece reflecting on your vision (Kapp (a)).

Autism symptoms can indeed be characterized by proxy information derived from our movements and associated sensations. Today, with the advent of the digital revolution, we can objectively quantify and track our movements and their consequential sensations over time. We can leverage the movements' natural biorhythms and augment them with therapies that naturally help improve our sensations in a timely fashion, while dampening pain and distress. Through innovative therapeutic approaches, we can integrate the peripheral contributions of our bodies in motion, allowing for the coherence of timing and coordination of our actions and their plans. This integration enables us to observe the beneficial effects of the rhythms of our movements and their sensations on various cognitive aspects of our actions. Along different dimensions, from head to toe, and different times in development, from infancy to adulthood, we can appreciate through our new RT how movements and their sensations help us regain our motor control and afford us more agency over daily living skills.

The 2025 RT *Autism: the movement (sensing) perspective a decade later*, comprises 28 articles by over 100 authors. They include one Opinion Piece, one Perspective article, one Review article, three Systematic Review articles, four Hypothesis and Theory articles, and 18 original research articles. The authors come from diverse backgrounds and perspectives, but they are united in their commitment to autism. They are scientists, parents, self-advocates, and entrepreneurs helping improve research by contributing to the funding and dissemination of scientific and technological advancements. Together, we aim to accelerate the translation of our science and technology into direct applications that can benefit autistic individuals, their families, and society by identifying novel methods for assessing movement, uncovering behavioral and neurobiological correlates of movement, and discovering effective and promising treatment options.

## Novel methods of assessing and detecting movement (sensing) differences in autism

Novel methods for detecting movement differences from infancy through adulthood are presented in this RT. In “*Infants on the move: bibliometric analyses of observational vs. digital means of screening infant development*” by Varkey et al., the authors survey the literature on important observational methods from Pediatrics that track very early motor neurodevelopment through the general movements theory. They flag differences that are identifiable much earlier than when basing assessment on social or emotional criteria, as these develop and stabilize much later. New digitized versions of such traditional observational methods are uncovered by this bibliometric study, pointing to a fast-growing literature that demonstrates the potential scalability of new technologies and the rapidly growing need for adopting clinically informed digital biometrics. In “*A comparative study on fundamental movement skills among children with autism spectrum disorder and typically developing children aged 7–10*” by Dong et al., we appreciate the importance of tracking motor issues as core symptoms of autism spectrum disorders, rather than as mere comorbidities secondary to social and communication differences.

In understanding how best to measure movement differences in the context of an autism diagnosis, Bermperidis et al. redefine the concept of socio-motor agency by balancing bodily motor autonomy from the bottom up with top-down motor control. This is done in the context of the type of social dance that emerges when two agents interact socially during the administration of the very test that defines autism for research purposes. The digitized version of the Autism Diagnostic Observation Schedule (version ADOS-2) is presented on the original research article “*Digital screener of socio-motor agency balancing motor autonomy and motor contro*l” Bermperidis et al.. Here, new analytical methods are offered to significantly reduce the test administration time from an hour to merely 10 min, making it less taxing on both the person receiving the diagnosis and the clinician administering and scoring the test. The work further demonstrates how the motor code can automatically differentiate males from females and through machine learning (ML) techniques, automate the process of diagnosing autism through brief socio-motor assays.

Automation in detecting motor differences using ML methods is also appreciated in the original research article “*Machine learning's effectiveness in evaluating movement in one-legged standing test for predicting high autistic trait*” by Ohmoto et al.. When combined with video, this popular balance test revealed specific features that enhance the automatic detection of autistic motor traits in the general population, with a focus on the shoulder and waist areas. Similarly, following the generality of movement differences across the human spectrum, the original research article entitled “*Level of autistic traits in neurotypical adults predicts kinematic idiosyncrasies in their biological movements*” by Lewis et al., uses kinematic analysis to reveal movement differences that influence socio-motor behaviors in general. The authors suggest that “autistic traits may intricately influence the movement expressions at the microlevel, highlighting the need for a more nuanced understanding of the potential endophenotypic characteristics associated with social movements in neurotypical individuals.”

Moving beyond whole-body movement measures, “*Decreased wrist rotation imitation abilities in children with autism spectrum disorder*” by Liu et al. tests an ecologically informed motor task that requires the imitation of wrist rotations using low-cost wearable inertial sensors. The study found shorter total wrist rotation time, lower rotation amplitude, and weaker rotation symmetry in autistic children, suggestive of fine motor movement differences in autism. Examining facial movements, Torres, Vero et al., present brief, fully automated and highly scalable assays to measure facial micro-motions in autistics and neurotypicals using apps in smartphones and tablets. “*Hidden social and emotional competencies in autism spectrum disorders captured through the digital lens*” Torres, Vero et al., opens new ways to more naturally, frequently and comfortably quantify social and emotional predispositions of autistic individuals by focusing on motor nuances that currently escape the naked eye. This original research article leverages the recent digital revolution to provide a unifying platform for studying natural biorhythmic motions recorded using simple, affordable, and ubiquitous means.

Further delving into the current issues that Technological advances address, the perspective piece entitled “*Unlocking autism's complexity: the Move Initiative's path to comprehensive motor function analysis*” by Good and Horn, provides a roadmap for Citizen Science in collaboration with academia and industry to accelerate the translation of the science to practical settings, while recognizing the nuances of behavioral exchanges that transpire largely beneath awareness and yet, seem to be the critical ingredient in fostering interpersonal connection. “The Move seeks to accelerate the integration of the expanding knowledge base into widespread practice. Deep, longitudinal, multi-modal profiling of individuals with autism spectrum disorder offers an opportunity to address gaps in current data and methods, enabling new avenues of inquiry and a more comprehensive understanding of this complex, heterogeneous condition.”

## Behavioral and neurobiological correlates of movement (sensing) differences in autism

The articles in this RT also demonstrate the associations between movement, the brain, and other elements of cognition or daily functioning. In “*Beyond words: an investigation of fine motor skills and the verbal communication spectrum in autism*” by Simarro Gonzalez et al., and “*The relation between specific motor skills and daily living skills in autistic children and adolescents*” by Skaletski et al., fine motor skills are shown to relate to speaking abilities and daily living skills in autism. In “*The relationship between executive function and the association of motor coordination difficulties and social communication deficits in autistic children*” by Gu et al., the commonly found association between motor skills and social communication was found to be partially mediated by executive function, highlighting the important intersection of movement, cognition, and social communication in this population.

In examining neurobiological correlates of hand grip strength, the original research article entitled “*Microstructural neural correlates of maximal grip strength in autistic children: the role of the cortico-cerebellar network and attention-deficit/hyperactivity disorder features*” by Surgent et al., provides important insights on the role of the cerebellum for feedback-based control, planning and prediction, so relevant to the notion of internal models for action. Importantly, this paper extends the issues in motor control from autism to other disorders of the nervous systems that co-exist today with the autism diagnosis, owing in part to the change from DSM IV to DSM-5 criteria. Specifically, we see an increase in the prevalence of motor and sensory issues in autism with the inclusion of attention deficit hyperactivity disorder (ADHD) in the ever-broadening spectrum. This adds to the spectrum other related movement disorders that accompany ADHD (*e.g.*, Tourette's syndrome and obsessive-compulsive disorders).

Finally, in “*Motor imagery in autism: a systematic review*” Gowen et al. raise awareness of the extensive psychological literature on mental chronometry and mental imagery as a possible avenue for investigating movement differences in autism. The piece highlights the importance of the methods and the need to develop and adapt new assays that could help us better understand MI in autistic individuals. Gowen et al. invite the development of full batteries for explicit and implicit MI tasks, an effort that would advance our understanding of elements of MI that may be affected in autism and the therapeutic potential of MI in these populations. Highly relevant to this proposition is the need to bridge mental imagery with physical exercise, as various new emerging therapies are based on physical movements that require their mental representations, planning, and rehearsals, mediated by the biorhythms of our multilayered, multifunctional motions.

## Novel approaches to movement (sensing) intervention/treatment

This RT provides insights into current and novel therapeutic approaches based on the movement (sensing) literature, examining evidence for the impact of physical exercise interventions as well as the theoretical basis for other therapies that tap into synchronized movements and involve dyadic and group interactions.

The influence of physical exercise with therapeutic value for improving movement-based feedback, enhancing social interactions, and communication are described across three systematic reviews. “*Analyzing the influence of physical exercise interventions on social skills in children with autism spectrum disorder: insights from meta-analysis*” by Koh provides evidence for the importance of physical exercise in autism specifically in helping with differences in social interactions and communication, while considering both age appropriateness and duration of the intervention. “*The impact of physical exercise interventions on social, behavioral, and motor skills in children with autism: a systematic review and meta-analysis of randomized controlled trials*” by Wang et al., points out the benefits of physical exercise for cognitive control, mental flexibility and overall social skills, with direct application to these differences in autistic children. They highlight evidence from the literature that supports the need for personalized interventions and emphasizes the importance of considering age as a key factor in designing outcome measures. The work also emphasizes the need for larger sample sizes and long-term follow-ups to confirm the generalization, permanency, and transfer of the beneficial effects from the therapeutic context to daily life. “*A network meta-analysis of the effect of physical exercise on core symptoms in patients with autism spectrum disorders*” by Li et al., compares the effects of different sports programs on symptomatology of autism described at the level of social interactions. Importantly, this systematic review of the literature underscores the selectivity of different types of exercise programs for enhancing specific aspects of social interactions and communication. These reviews are complemented by findings of the beneficial effects of physical exercise which extend from individual to group sports in the research article entitled “*Effects of group sports activities on physical activity and social interaction abilities of children with autism spectrum disorders*” by Xing et al..

In physical exercise and other social interactions, we can appreciate the role of dyadic movements in the self-discovery of goals that improve social timing and communication. New emerging therapies that rely on the natural rhythms of bodies in motion may enhance bodily awareness through cooperative settings where the therapist guides the person without enforcing a top-down, preconceived plan. Using different time scales that may range from those mediated by rich musical exercises in Neurological Music therapy (NMT), to slow, interactive rhythms sensed together but set by the autistic person's natural pace, these therapies hold promise as they are adapted from general movement disorders to autism settings. Already enjoying great popularity in other fields, they bring new elements to the autism realm. The therapist enables spontaneous resonance of the two bodies in motion and self-discovery of mutual empathy, promoting self-confidence and better coordination and control of their integrated rhythms. This shared give-and-take of rhythmic energy between two people, across therapeutic styles, positions the new emerging therapeutic methods as promising candidates for the development of healthier social mind-body relations in general. In autism, they empower the person's autonomy and bring a new level of emotional wellbeing that current behavioral therapies in the US severely lack. Further work under controlled conditions may be necessary to fully understand the optimal use of such interventions and maximize their benefits.

Indeed, the community delivering the most prevalent intervention for autism in the US (enjoying medical insurance coverage) is asking for change, seeking to update their behaviorist approaches from the 1950s. Rooted in Skinner's animal conditioning principles, they lack scientific proof demonstrating their efficacy in human neurodevelopment. The rather archaic methods, so prevalent in autism, are finding resistance within their own accredited therapists who report an increasing sense of futility and frustration about what they do. The Board-Certified Behavioral Analysts (BCBAs) in the state of New Jersey and across the United States are themselves scrutinizing the practice of Applied Behavioral Analysis (ABA). Graduates of the therapy that is offered by default across the schools in the US who reach 21 years of age and exit the school system report symptoms of PTSD and distress. Meanwhile, the therapists themselves report that they would welcome new digital technologies to better serve their clients in a fast-changing landscape of therapies under a business model that is uniquely failing their clients but has little incentive to improve. A new generation of therapies in the US that do not receive medical insurance coverage yet are persuading families of their effectiveness and organically pulling them away from ABA. Torres, Twerski et al., present the results of surveys that the BCBAs themselves designed and deployed, revealing the dawn of a new era in autism treatments. This is presented in the original research article entitled “*The time is ripe for the renaissance of autism treatments: evidence from clinical practitioners*” Torres, Twerski et al..

“*A handbook for Rhythmic Relating in autism: supporting social timing in play, learning and therapy*” by Daniel et al., details new ways to support play, learning, and therapy with young autistic children, unconventional communicators, and autistic people who have additional learning needs. By following the form of vitality in movement created through reciprocal play, Daniel et al. provide core foundations for practitioners and parents to help build rapport and connection by sharing meaning in movement.

LaGasse et al., present a hypothesis theory piece entitled, “*Rhythm and music for promoting sensorimotor organization in autism: broader implications for outcomes*.” This includes first-hand experiences by a self-advocate that alerts us to the beneficial elements of this form of therapy. Richard Williams, Hurt-Thaut et al., provide evidence for pre- *vs*. post-NMT improvements of motor control in “*Improved motor skills in autistic children after three weeks of neurologic music therapy via telehealth: a pilot study*” Richard Williams, Hurt-Thaut et al.. Importantly, in this original research article, the authors successfully use telehealth as a promising new avenue to scale the research and practices related to NMT. Broadly in support of the use of auditory cueing during NMT, Richard Williams et al. also provided evidence that augmented auditory feedback decreased motor variability in autistic participants in “*Auditory feedback decreases timing variability for discontinuous and continuous motor tasks in autistic adults”* Richard Williams, Tremblay et al..

Further beneficial aspects of leveraging the biorhythmic activities of the nervous system are presented in a research article entitled “*Evidence of mutual non-verbal synchrony in learners with severe learning disability and autism, and their support workers: a motion energy analysis study*” by Glass and Yuill. Contrary to traditional views, the authors here highlight the capacity of high-support autistics and non-speakers to facilitate interpersonal motor synchrony while learning through rhythmic interactions, a theme also supported in “*Bridging the gap: fostering interactive stimming between non-speaking autistic children and their parents*” by Chen. In this original research article, the author tracks three non-speaking children and their mothers during interactive rhythmic therapy, which is mediated by touch, music, and the self-generated stimming motions of the children. By promoting the children's autonomy, they anchor the starting point in stimming and expand their gestural repertoire from there, significantly improving interactions with their mothers. Through sounds coupled to their bodily motions, they open the repertoire of communicative gestures, co-creating sensory experiences in multisensory environments. This paper demonstrates the therapeutic and pedagogical benefits of embracing various modes of communication, particularly when they leverage the autistic person's self-discovered movement-sensing compensatory strategies.

Music and the rhythms of one's own body shared with the rhythms of other bodies in motion promote better timing and coordination, often enhanced by touch, mediated by sounds, and a mixture of spontaneous and deliberate actions. These improvements, which lead to better social timing, are systematically achieved and amplified by another family of therapies featured by Baniel et al. in “*From fixing to connecting—developing mutual empathy guided through movement as a novel path for the discovery of better outcomes in autism*.” [26] In this hypothesis theory article, the authors introduce two well-known and successful therapies, the Feldenkrais Method and the Anat Baniel Method^®^ NeuroMovement^®^. These approaches, already popular in stroke rehabilitation, Parkinson's disease, acquired dystonia (in musicians), and cerebral palsy, among other movement disorders, aim to enhance bodily awareness, as participants improve their sense of kinesthetic reafference. This critical, yet subtle aspect of our motor control and coordination is often neglected owing to its subliminal nature.

The above body of work fosters a full awareness of possible ways to engage the person's sensations and the differentiation of self-generated motions, in the context of interacting one-on-one with the practitioner and as a member of a group. Practiced in dyads and in groups, these various forms of therapies offer new ways to reinforce the biorhythmic visuospatial body maps and bring awareness of dormant body parts to the person's brain through fluid movement. By feeding new movement information to the brain at a conscious level, and by doing so at an adaptable, comfortable pace, the brain learns to anticipate and track the outcomes of the movements that it self-generates and plastically remaps the representations of bodies in motion. It is in this spatial information of one's own proprioception (the internally sensed bodily postures) that one can then anchor the timing of the consequences of one's own motions. These in turn, align with the timings of the motions of others moving in the space that is external to one's own moving body. In NMT, sound-informed external rhythms help the person synergize the body's abundant degrees of freedom and optimize the motions. Timely physical interaction with others during exercise also helps with the rhythmicity of the motions. And in the Feldenkrais Method and the Anat Baniel Method^®^ NeuroMovement^®^, synchronicity spontaneously emerges from within while learning to bring focused attention to the movements' biorhythms. Together, these therapies adapted to autism bring a new era in the treatment of nervous system functions, both during neurodevelopment and throughout adulthood. Transferring such benefits to other learning and cognitive domains can also be appreciated across several original research articles in the RT.

As basic research results pointing out movement differences in autism try to make their way into medical practice, many obstacles emerge that impede progress despite the growing body of scientific evidence supporting the need to consider motor issues. Key points related to primary care are explained in the original research article entitled “*Barriers and facilitators to primary healthcare encounters as reported by autistic adults: a qualitative study*” by Stein Duker et al.. The work describes barriers and facilitators to primary care encounters as reported by autistic adults, providing nuanced accounts from autistic adults themselves and highlighting their unique perceptions of barriers and facilitators to their interactions with the healthcare systems. In the very words of the authors, “These results offer valuable insights for improving the accessibility and quality of care for autistic people, many of which are practical, low/no cost, and easy to implement. Strategies also emphasized the diversity of experiences and preferences for autistic patients, highlighting the importance of tailoring accommodations in the primary care setting.”

Altogether, the articles in this RT provide new insights into movement measurement, associated constructs, and therapeutic pathways. In the last article, we come full circle with Dr. Kapp (b), who opened our RT honoring Professor Anne Marie Donellan, and highlights “*Sensory–movement underpinnings of lifelong neurodivergence: getting a grip on autism*” Kapp (b). Through the lens of self-advocacy and science, Kapp (b) offers a genuine, personal account of what movements and their sensations can do for all. Indeed, our Research Topic is signaling an Autism Renaissance that includes, at its core, the movement-sensing perspective.

